# Dielectric properties of complex magnetic field induced states in PbCuSO_4_(OH)_2_

**DOI:** 10.1038/s41598-017-04752-z

**Published:** 2017-06-30

**Authors:** T. Mack, A. Ruff, H.-A. Krug von Nidda, A. Loidl, S. Krohns

**Affiliations:** Experimental Physics V, Center for Electronic Correlations and Magnetism, University of Augsburg, 86135 Augsburg, Germany

## Abstract

Spin spirals, which coexist with collinear spin order in linarite PbCuSO_4_(OH)_2_, indicate electrical polarisation textures of spin-multipolar phases. We derive experimental evidence by a detailed investigation of the magnetic-field dependent dielectric and electric polarization properties at low temperatures. Linarite exhibits a quasi-one-dimensional frustrated S = ½ spin chain, which forms 3D spin-spiral order in zero magnetic field for T < 2.85 K. Recently, due to the monoclinic lattice of linarite with CuO_2_ ribbon chains, complex magnetic field induced states were found. These spin-multipolar phases, which compete with spin-density waves at low magnetic fields, exist in close vicinity to the transition from the spin spiral into field induced spin polarized state. Via antisymmetric Dzyaloshinskii-Moriya interaction spin-driven ferroelectricity develops in the spin-spirals state. Via electric polarization measurements this allows to prove the transitions into complex magnetic field induced phases. Thorough analyses of the temperature and magnetic field dependent dielectric properties of a naturally grown single crystalline sample provide a detailed (T,H) phase diagrams for the three different crystallographic directions.

## Introduction

At low temperatures frustrated low-dimensional spin chains exhibit various complex magnetic phases. This originates from competing ferromagnetic (FM) and antiferromagnetic (AFM) nearest neighbour interactions^1^. In PbCuSO_4_(OH)_2_ the spin chains of Cu^2+^ spin ½ ions are realized by CuO_2_ ribbons of edge-sharing CuO_4_ square planes. This leads to nontrivial magnetic structures arising from competing FM Cu-O-Cu (J_1_) and AFM Cu-O-O-Cu (J_2_) exchange interactions. Depending on the frustration parameter α = J_2_/|J_1_| various ground states are predicted^2, 3^, especially if quantum fluctuations and local anisotropy are taken into account^4^. In a classical spin model a spin spiral is formed for α > ¼^5^. But, tuning the α parameter, instabilities of spiral states like their vector chirality can result in various spin multipolar phases (SMP), as quadrupolar bond-nematic state (p = 2)^6^ or multi magnon-bound states (p > 2)^1^. The p-values denote finite elementary magnetization steps as a function of magnetization and frustration^7^. For the low magnetization regime, in the ordered state (T < 2.85 K) only spin-spirals exist. On increasing magnetization, which is still slightly below saturation (order of 4 to 6 T), SMPs up to p = 9 evolve, depending on α and the applied magnetic field^1^. These SMPs and the spin-spiral state can coexist in close vicinity to the SMP transitions. The spin spirals are often accompanied by an electrical polarization due to the presence of tilted spins breaking the inversion symmetry, which is a prerequisite for ferroelectricity^8–10^. For quasi one-dimensional spin-1/2 chain systems as LiCuVO_4_^11–13^, LiCu_2_O_2_^14^ and PbCuSO_4_(OH)_2_^15, 16^ this ferroelectric (FE) polarisation were ascribed to a spin-driven improper FE effect at the three dimensional AFM ordering temperature^17–20^. The close coupling of magnetic order and electric polarization allows to switch the polarization by a magnetic field and to control via an electric field the spin helicity^21^. Detailed magnetic field dependent dielectric and polarization studies revealed these interesting multiferroic properties in LiCuVO_4_^11, 13, 21, 22^. We performed similar measurements for PbCuSO_4_(OH)_2_ to gain insight to the close coupling of magnetic and electric order, especially in the vicinity of the magnetic saturation field. Here, the electric component of the spin spiral is used as measure to determine SMPs and their possible polar textures.

Linarite crystallizes in the monoclinic P2_1_/m structure leading to CuO_4_ plaquettes, which form Cu-ribbons^23–25^. In zero magnetic fields the spin spiral is established in a plane spanned by crystallographic *b*-direction and by 27° tilted *a*-direction below 2.85 K. Magnetic fields up to 10 T induce at least five different magnetic phases^26, 27^. In addition, an unusual thermodynamic phase has been found for the *b*-direction slightly above the long-range magnetic order^1^. In this case, which is close to the saturation field, fluctuations hamper the primary spin order while the SMP persists. The experimental evidence for SMPs with p > 2, which may compete with exotic longitudinal collinear spin-density waves (SDW_p_), was given by Willenberg *et al*.^1^. Even multi-magnon bound states reaching p = 9 are achieved by tuning the frustration parameter in the vicinity of the magnetic saturation field. Above magnetic saturation in fields of m_s_ > 6 T, field induced spin-polarized states evolve. Here, the critical fields depend on the crystallographic directions where the magnetic field is applied. Interestingly, the saturation fields are by a factor of 5 smaller than for LiCuVO_4_^22^. This allows to investigate these complex magnetic phases in fields up to 10 T.

In this paper, we study in detail the electrical polarization properties of linarite as function of magnetic field and temperature. For LiCuVO_4_^13^, which is a prototypical example to test for switching of electrical polarization via a magnetic field, the polarization **P** is evolved only, if the vector product of spin spiral axis **e** and propagation of the spin spiral **Q** is finite, i.e. P ∝ *e* × Q^8^. In contrast, linarite was ascribed to show no rotation of the spiral axis **e** under an applied magnetic field due to its monoclinic structure^1^. However, we provide a thorough dielectric characterization as function of magnetic fields applied in *a*, *b* and *a*_⊥_-direction and reveal the correlation of magnetic and electrical order. In the present experiment only *a* and *b* directions are well defined with respect to the external fields. Due to fact, that the crystal structure is monoclinic, the *c* direction of this sample is not well known. At T_N_ a clear dielectric anomaly indicates the FE transition. This confirms the multiferroic nature of linarite^16^. Further significant dielectric signatures are found depending on crystallographic direction, applied magnetic fields and temperature. These transitions are discussed in terms of remnant switchable spin spirals and electrical polarization texture of SMPs.

**Figure 1:**
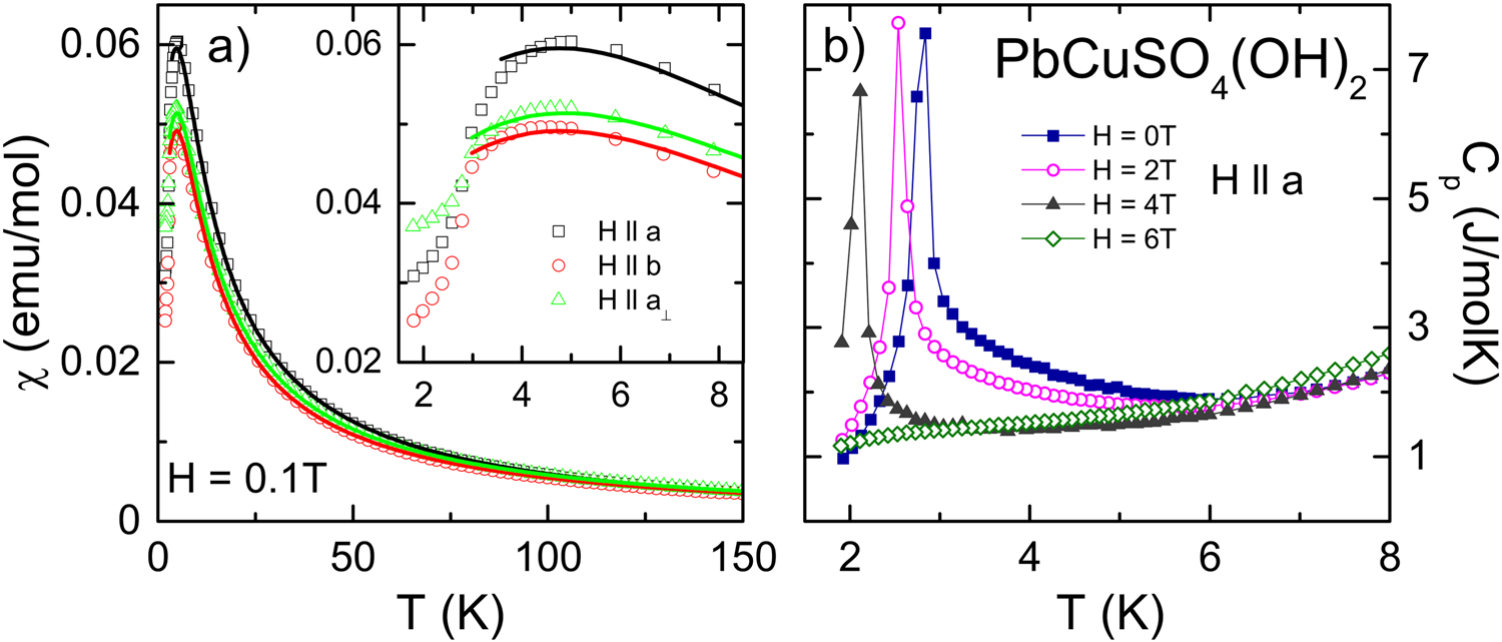
Magnetic susceptibility and specific heat measurements. (**a**) Temperature dependence of magnetic susceptibility χ of a PbCuSO_4_(OH)_2_ single crystal with magnetic fields H = 0.1 T along *a*, *b* and *a*_⊥_ directions. The lines represent fits with numerical Bonner-Fisher approximation. The inset shows a magnified view for T ≤ 9 K. (**b**) Temperature dependence of specific heat measured of a PbCuSO_4_(OH)_2_ single crystal with magnetic field along *a* direction for various applied magnetic fields.

We prove the sample quality of a natural grown single crystal from a mine in Lavrion (Greece) performing temperature dependent magnetic susceptibility χ (Fig. 1a) and specific heat measurements *c*_*p*_ (Fig. 1b). The magnetic susceptibility χ was analysed along *a*, *b* and *a*_⊥_ crystallographic directions in an applied magnetic field of H = 0.1 T. A distinct peak anomaly shows up around 5 K for all directions, followed by a step-like decrease at T = 2.85 K (inset of Fig. 1a). The latter denotes the antiferromagnetic ordering and is in good agreement with previously published results from linarite crystals of different origins^1, 15, 27^. The lines represent a reasonable fit of χ using a numerical approximation of an isotropic linear one-dimensional antiferromagnetic spin ½ chain^28, 29^. From these fits we determine an average antiferromagnetic exchange coupling of J/k_B_ = 3,7 K. The high temperature Curie-Weiss behaviour signals a Curie-Weiss temperature close to 0 K indicating competing nearest and next nearest neighbour exchange. The inset of Fig. 1a indicates slightly anisotropic *g*-values with maximum value for H || *a* in fair agreement with literature^16, 30^. Figure 1b show specific heat measurements along *a* direction for various applied magnetic fields. The peak anomaly in *c*_*p*_ at T_N_ and its magnetic field dependence originating from long-range spin spiral formation confirm the literature^1, 15, 27^. Both measurements evidence reasonable sample quality, which allow the investigation of electrical polarization of the complex magnetic phases close to T_N_.

## Spiral spin configuration

**Figure 2:**
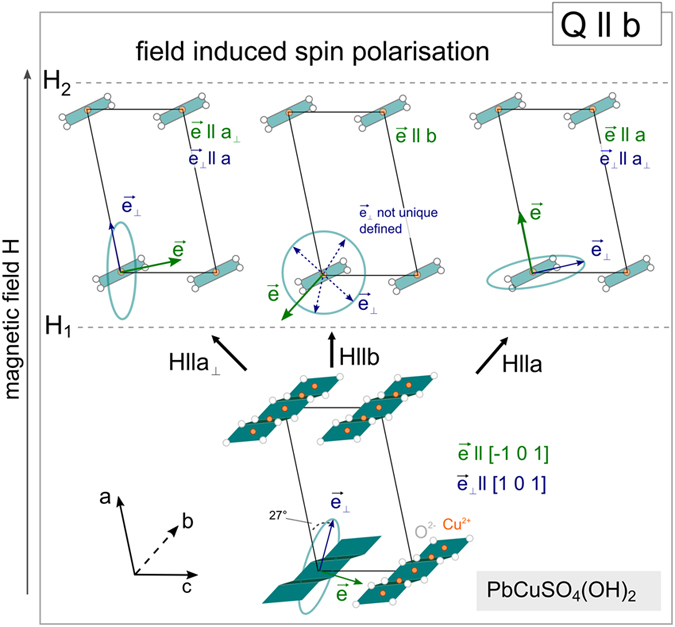
Schematic sketch of the spin configurations in the magnetically ordered phases of PbCuSO_4_(OH)_2_ as function of external magnetic field. The switching of the spin spiral for the different magnetic field directions is indicated in analogy to LiCuVO_4_. In the ground state the elliptical helix shows a component in *b*-direction while the other component is forming an angle of 27° with the *a*-axis (spiral axis *e* almost [−1 0 1]). *H*_*1*_ and *H*_*2*_ indicate phase boundaries between the different magnetic phases.

In analogy to LiCuVO_4_^13^, Fig. 2 schematically shows the ground state of the spiral spin configuration as function of magnetic field. For clarity reasons only the magnetic CuO_4_ plaquetts in ribbon structure are depicted. The spin configuration of the ground state has been systematically characterized by neutron diffraction^26^, magnetization^22, 27^, NMR^1^ and specific heat measurements^27^. The spin spiral exhibits a modulation vector Q parallel to the *b* direction and lies in a plane spanned by *b* and a vector tilted by 27° from *a*. Thus, the spin spiral axis *e* is almost parallel to crystallographic [−1 0 1] direction. In this case inverse Dzyaloshinskii-Moryia interactions (DMI) or spin currents induce an electric polarization in *e*_⊥_, i.e., parallel to [1 0 1] direction. In contrast to ref. 1, we assume a rotation of the spin-spiral axis *e* at a critical field H_1_ into the applied external magnetic field direction, while the modulation vector is unchanged. Between H_1_ and H_2_, namely for H || *a*_⊥_ the polarisation is along *a* and for H || *a* along the *a*_⊥_ direction. Here, we use *a*_⊥_, which is perpendicular to *a*, that means a direction counter clockwise tilted by 13° from *c* towards *a*. In case of H || *b* above H_1_, the spiral axis is along the modulation vector, which prevents polarisation via P ∝ *e* × Q. However, in the vicinity of H_1_ instabilities of the spin spirals result in SMPs^1^. A fully spin-polarized state evolves with a collinear spin structure for H > H_2_. For linarite the upper critical field H_2_ depends on the crystallographic direction and is of the order of 6 to 8 T.

## Magnetic field along *a*_⊥_-direction

**Figure 3:**
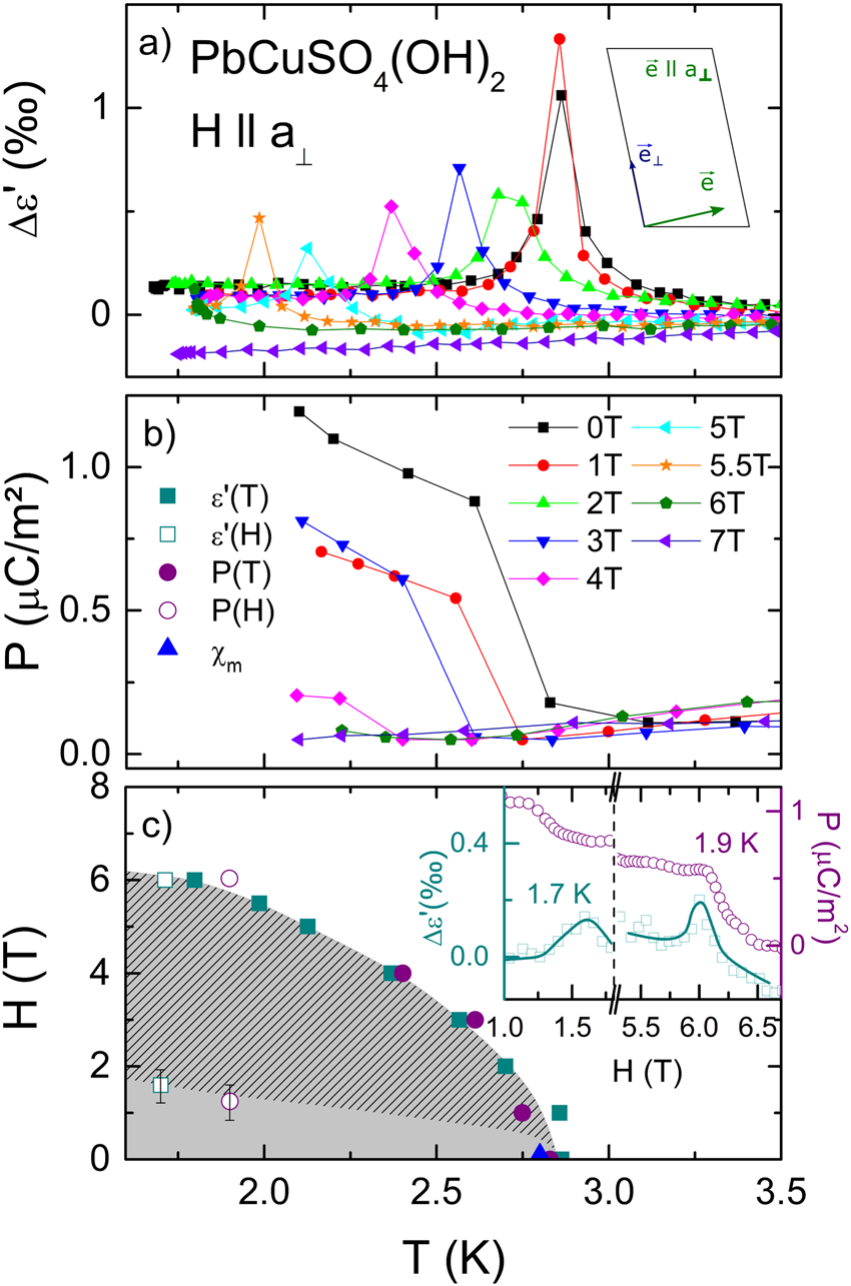
Dielectric properties at 300 Hz with E || *a* of a PbCuSO_4_(OH)_2_ single crystal with magnetic field along the *a*_⊥_ direction. (**a**) Temperature dependent dielectric constant for various magnetic fields. The dielectric constant is normalized to a constant value and deviations are shown in parts per thousand (‰). The inset illustrates the spin configuration between *H*_1_ and *H*_2_. (**b**) Temperature dependent electrical polarization performed for different magnetic fields, with a poling field E_pol_ = 2 kV/cm. (**c**) (*T*,*H*) phase diagram constructed using results from the present work; the grey shaded area indicates the rotated spin spiral state. The inset shows magnetic field dependent dielectric constants and polarization at 1.7 and 1.9 K, respectively (lines are drawn to guide the eye).

**Figure 4:**
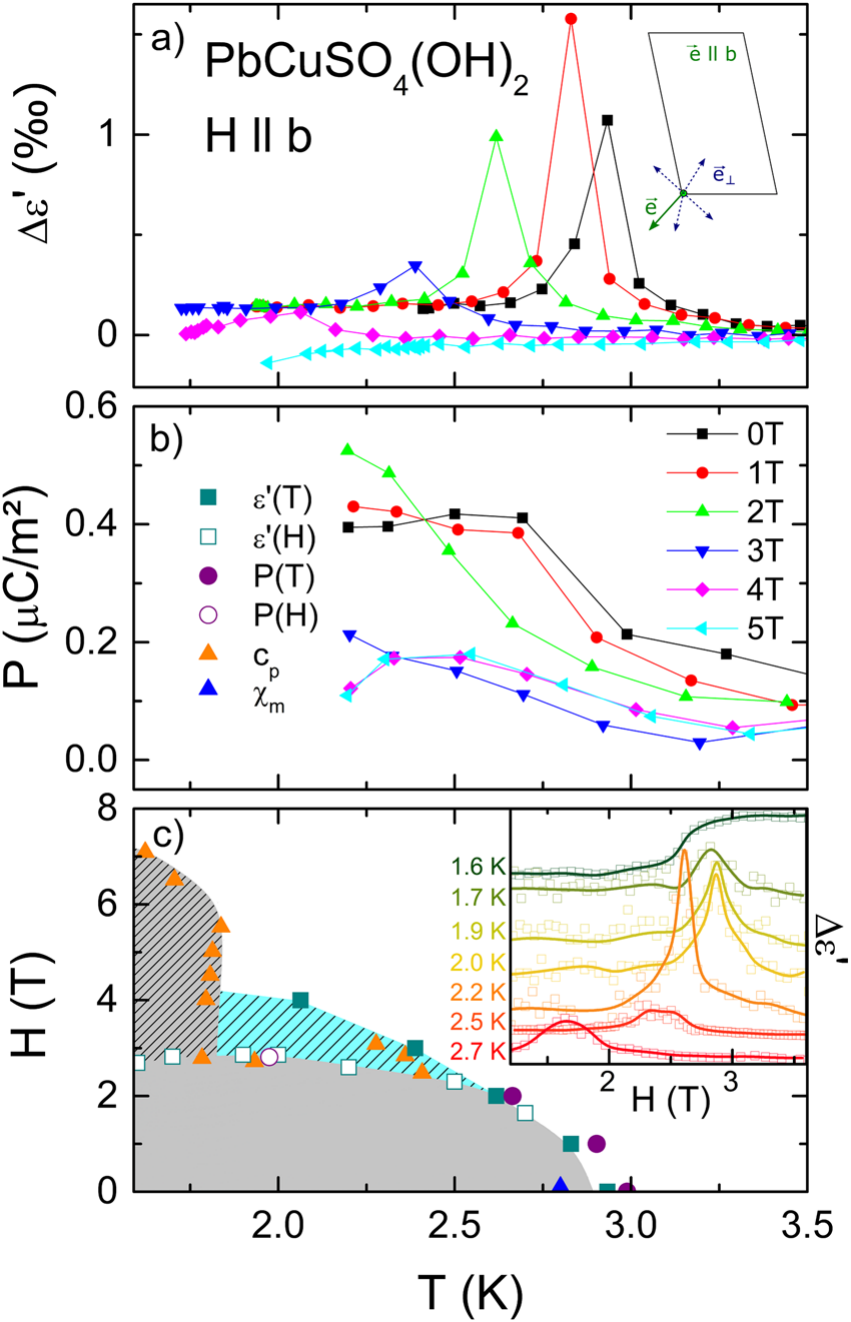
Dielectric properties at 300 Hz with E || *a* of a PbCuSO_4_(OH)_2_ single crystal with magnetic field along the *b* direction. (**a**) Temperature dependent dielectric constant for various magnetic fields. The dielectric constant is normalized to a constant value and the deviation is shown in ‰. The inset illustrates the spin configuration for H > *H*_*1*_. (**b**) Temperature dependent electrical polarization performed for different magnetic fields with a poling field E_pol_ = 2 kV/cm. (**c**) (*T*,*H*) phase diagram with results from the present work and from ref. 27 (c_p_). The inset shows magnetic field dependent excess dielectric constant, as observed from 1.6 to 2.7 K (lines are drawn to guide the eye).

**Figure 5:**
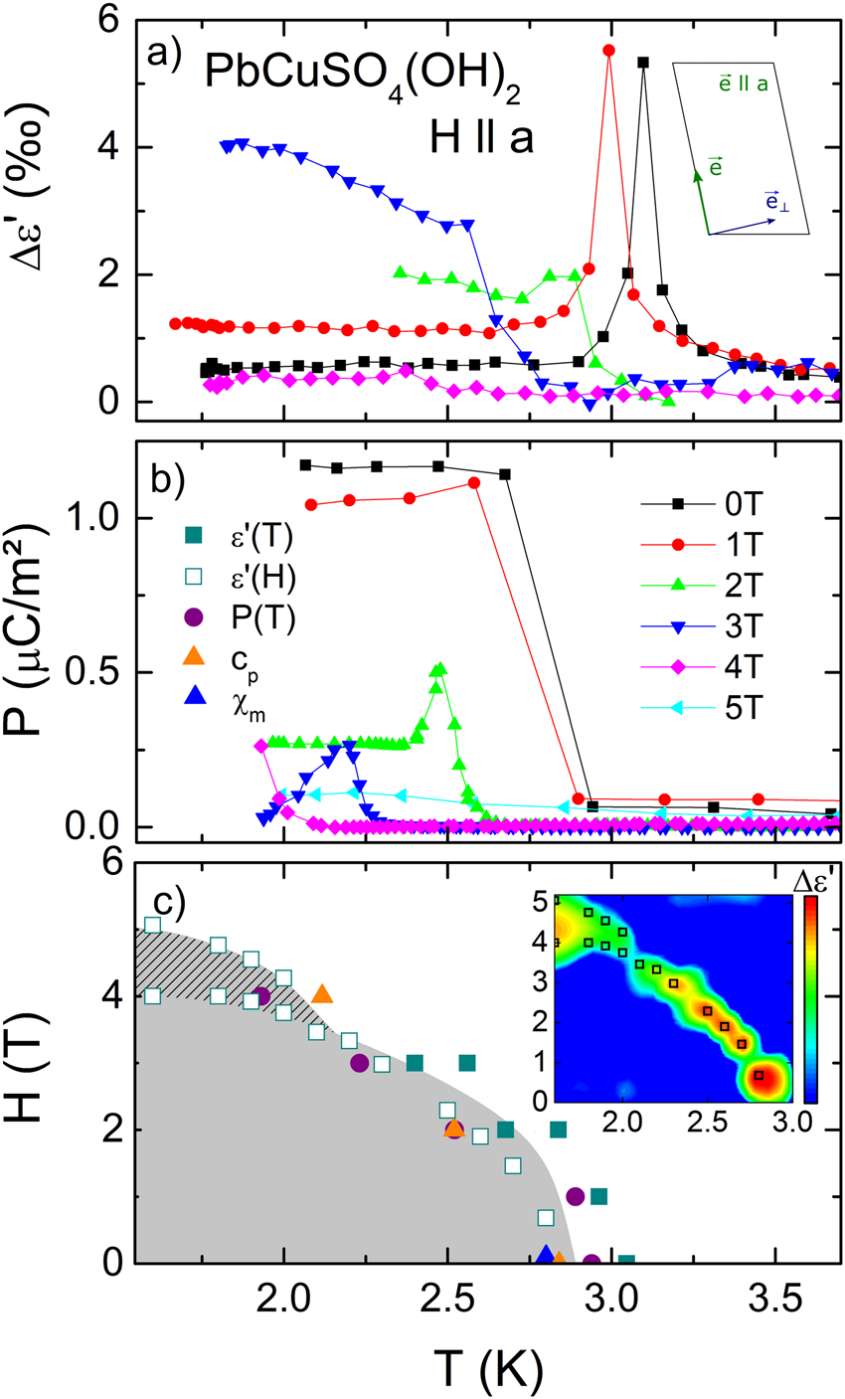
Dielectric properties at 300 Hz with E || *a*_⊥_ of a PbCuSO_4_(OH)_2_ single crystal with the magnetic field along the *a* direction. (**a**) Temperature dependent dielectric constant for various magnetic fields. The dielectric constant is normalized to a constant value and the deviation is shown in ‰. The inset illustrates the spin configuration for H > *H*_*1*_. (**b**) Temperature dependent electrical polarization performed for different magnetic fields with a poling field E_pol_ = 2 kV/cm. (**c**) (*T*,*H*) phase diagram, which results from the present work. The inset shows a heat map of the temperature and magnetic field dependent deviations in dielectric constant, the peak positions are denote by the open squares.

As indicated in Fig. 2 three cases above H_1_ are of interest: i) H || *a*_⊥_ in Fig. 3, ii) H || *b* in Fig. 4 and iii) H || *a* shown in Fig. 5, respectively. In Fig. 3a we present the temperature and magnetic field dependent deviations of the dielectric constant in parts per thousand (‰). This was calculated using the static dielectric constant in zero magnetic field at T = 3.5 K. The external magnetic field up to 7 T was applied along the *a*_⊥_-direction and the dielectric properties as well as the polarization measured in *a*-direction. Significant peak anomalies in Δε’ emerge for H < H_1_ ≈ 2 T at T_N_ ≈ 2.85 K. The dielectric constant and dielectric loss (not shown) reveal no frequency dependence of the peak anomaly, which implies an improper FE phase transition. The peak anomaly in Δε’ (Fig. 3a) and the step-like decrease in P shift for H_1_ < H < H_2_ ≈ 6 T to lower temperatures. For 4 T the transition temperature in Δε’ is at 2.4 K. In addition the peak height of Δε’ and the magnitude of the polarization decrease by a factor of two by increasing the magnetic field up to 4 T. This reduced polarisation originates from the rotation of the spiral spin axis from [1 0 1] into *a*-direction. No dielectric anomalies were found for H > H_2_, pointing towards the absence of a spin-spiral and a possible existence of a non-polar fully spin-polarized state. Similar behaviour was observed in the magnetic phase diagram as shown in ref. 13. The results of the dielectric and polarization measurements are summarized as a qualitative (T,H)-phase diagram in Fig. 3c. Our results are in good agreement with the magnetic phases determined in ref. 27. The onset of the polarisation is about 0.2 K lower compared to the other measurements. This originates from a thermal hysteresis of the pyrocurrent measurements, where the signal is measured on heating in contrast to the other analyses that were performed on cooling the sample. Magnetic field dependent measurements at 1.9 and 1.7 K reveal peaks in Δε’ and steps in P at both critical fields H_1_ and H_2_ (inset of Fig. 3c). This verifies on the one hand the rotation of the spiral spin axis at H_1_ and on the other hand denotes the upper critical field for ferroelectricity at H_2_. However, compared to LiCuVO_4_ the monoclinic structure of linarite hampers the switching of the improper FE order and so, its overall polarisation is significantly lower.

## Magnetic field along *b*-direction

In the vicinity of H_1_, for H || *b* the occurrence of dielectric and polarization anomalies probably results from SMPs, which – as discussed in the following – originate from instabilities of the spin spirals. Figure 4a shows the temperature dependent deviation of the static dielectric constants in applied magnetic fields up to 5 T. For H < H_1_ ≈ 3 T^27^, the peak anomalies indicate that the temperature of the FE phase transition decreases for H > 1 T. Interestingly, even for H > H_1_ the peak in Δε’ is not suppressed and instead shifted for H = 4 T to 2 K. Figure 4c shows a qualitative (T,H) phase diagram. For H > H_1_ Δε’(T) follows rather the phase boundary of the blue-dashed area, which denotes the SMP, than the one of the spin spirals (grey area). The SMP boundary has been identified from NMR studies^1^ and specific heat measurements^27^. The latter ones are depicted in Fig. 4c as orange triangles and confirm the phase boundaries (c.f. phases I, II, IV and V of refs 1 and 27) revealed by dielectric, pyrocurrent and magnetocurrent measurements. The results of dielectric spectroscopy as an indirect measure of the spin spirals indicate a polar texture of a SMP, where close to the saturation field instabilities of spin spirals persist. In Fig. 4b the onset of polarisation and the polar strength are shown, which are even for H < H_1_ close to the resolution limit, that impedes a correlation of polar strength to distinct complex magnetic order (p ≥ 2). However, the qualitative onset of a steplike feature of the polarisation clearly denotes for H < H_1_ the phase boundary of the spin spiral state (grey shaded areas of Fig. 4c). For H > 3 T the polar spiral state shows up below the accessible temperature range for pyrocurrent measurements.

To obtain quantitative information for the SMP, we conducted magnetic field dependent measurements of dielectric properties for T < 2.7 K. The inset of Fig. 4c shows the deviations of static dielectric constants for T < T_N_. Peak anomalies clearly indicate improper FE transitions for 2.85 K > T > 1.7 K. These measurements were also performed for increasing and decreasing magnetic fields. A hysteretic behaviour in the peak anomalies of ε’(H) is present (not shown) and less than 0.1 T, which is in good agreement with magnetocaloric–effect measurements discussed in ref. 27. These peaks perfectly describe the spin-spiral phase boundary, which was determined by ac magnetic susceptibility measurements (Fig. 4c, grey area)^27^. Interestingly, the peak in Δε’(H) at 1.7 K smears out and especially at 1.6 K it rather shows up as a step than a peak anomaly. This is explained by entering the phase coexistence of a collinear spin arrangement and another helical phase^1, 27^. The latter one is depicted in Fig. 4c as grey-dashed area at low temperatures. But, field-dependent dielectric measurements reveal no significant transitions of the SMP into a field induced spin-polarized state. It seems, that only Δε’(T) for H > H_1_ is sensitive to a polar order resulting from instabilities of various spin spirals within the SMP. This further indicates that the observed polarization is a function of magnetic and thermal treatment and only detectable by dielectric measurements, when starting in the paramagnetic disordered state above T_N_. The anomalies of Δε’(T) for H > 2 T are then related to the polar moment of a locally persisting spin spiral as a consequence of fluctuations within the frustrated spin chain system.

## Magnetic field along *a*-direction

Figure 5 shows the results of dielectric and polarization measurements for E || *a*_⊥_ and H || *a*. For this case we expect a flip of the spin spiral axis from *e* || [−1 0 1] into crystallographic *a* direction at H_1_ ≈ 3.5 T. As shown in Fig. 2, this represents the “switching” case of the polarization P, which is aligned along [1 0 1] for H < H_1_ and turned into *a*_⊥_ direction for H_1_ < H < H_2_ ≈ 5 T. For the ground state of the spin spiral, P and *a*_⊥_ have an angle of about 63° allowing for pronounced signatures of improper FE order even for H < H_1_. This shows up as Δε’-peaks in Fig. 5a and steps in the polarization (Fig. 5b). Increasing the external magnetic field, leads to a drop in polarization down to 0.3 µC/m^2^ for 3 T and even lower for 4 T, finally vanishing for 5 T. The latter one is accompanied by a diffusive behaviour in Δε’, where a smeared out peak at 3 K followed by a slight increase is observed. In addition, measurements at 3 T reveal this type of dielectric anomaly, too. This is explained either in terms of a spin flip of *e* above H_1_ or by instabilities of the spin spirals close to the saturation fields. Both mechanisms lower the polarization in *a*_⊥_ direction. Figure 5c depicts the resulting qualitative (T,H) phase diagram, where the grey dashed area denotes the anomalous phase. To obtain quantitative information about the transition temperatures, we conducted magnetic field-dependent dielectric measurements for T < 3 K. Δε’(H,T) clearly reveals a structure consisting of a broad and a sharp peak for temperatures from 1.6 K to 2 K. Above 2 K only one distinct peak-feature persists. This is illustrated as a heat map (inset of Fig. 5c). The green and red areas denote local maxima in Δε’(H), which can be explained by two consecutive improper FE transitions: polarization along *a*-direction for H < H_1_ followed by the switched polarization in *a*_⊥_-direction or instabilities of spin spiral state below H_2_. Finally, no FE order persists above H_2_.

## Summary

We investigated in detail the multiferroic properties of natural grown single crystalline linarite. This material exhibits a rich variety of complex multiferroic phases for T < T_N_. Superior to other spin-chain compounds, like LiCuVO_4_, the saturation fields are less than 10 T. Via magnetic field dependent dielectric and polarization experiments we found clear evidence that the improper ferroelectric transitions follow the P ∝ *e* × Q relation. This allows to determine distinct magnetoelectric (T,H)-phase diagrams for the different crystallographic directions. Moreover, as an indirect measure, dielectric spectroscopy also provides experimental evidence of spin-spiral instabilities of the SMPs. Finally, pyroelectric and dielectric investigations down to 0.25 K are necessary to clarify possible multiferroic interactions in further complex magnetic phases of linarite.

## Methods

A naturally grown PbCuSO_4_(OH)_2_ single crystal originating from a mine in Lavrion, Greece was investigated. The crystal has an approximate size of 1.9 × 1.3 × 0.5 mm and exhibits a monoclinic structure with space group P2_1_/m. It is oriented in (100) direction. Magnetic susceptibility and specific heat measurements were performed in Quantum Design MPMS and PPMS systems. Dielectric measurements for frequencies between 300 Hz and 10 kHz were done using an AH2700A capacitance bridge. The FE order was examined by pyroelectric and magnetoelectric current measurements. Therefore a Keithley 6517 A electrometer was employed, which was also used to apply a poling field of the order of 2 kV/cm during the cooling process of the sample. Subsequently to this poling, the pyrocurrent was detected without applied electric field on heating the sample. A Quantum Design PPMS and an Oxford cryomagnet were used for magnetic fields dependent measurements up to 10 T in the temperature range from 1.5 to 300 K. In order to perform dielectric measurements along different crystallographic directions silver-paint contacts were applied to the plate-like crystal in two geometries: a sandwich build-up to prove the polarization along the *a* axis and a cap-like shape covering two opposite ends to examine the polarization along *a*_*⊥*_ direction. All data of temperature dependent measurements, except the pyrocurrent data, were recorded while cooling the sample. The temperature dependent features show a thermal hysteresis of about 0.2 K.
